# Therapeutic Targets for Treatment of Heart Failure: Focus on GRKs and β-Arrestins Affecting βAR Signaling

**DOI:** 10.3389/fphar.2018.01336

**Published:** 2018-11-27

**Authors:** Supachoke Mangmool, Warisara Parichatikanond, Hitoshi Kurose

**Affiliations:** ^1^Department of Pharmacology, Faculty of Pharmacy, Mahidol University, Bangkok, Thailand; ^2^Department of Pharmacology and Toxicology, Graduate School of Pharmaceutical Sciences, Kyushu University, Fukuoka, Japan

**Keywords:** β-adrenergic receptor, β-arrestin, G protein-coupled receptor kinase, heart failure, β-blocker

## Abstract

Heart failure (HF) is a heart disease that is classified into two main types: HF with reduced ejection fraction (HFrEF) and HF with preserved ejection fraction (HFpEF). Both types of HF lead to significant risk of mortality and morbidity. Pharmacological treatment with β-adrenergic receptor (βAR) antagonists (also called β-blockers) has been shown to reduce the overall hospitalization and mortality rates and improve the clinical outcomes in HF patients with HFrEF but not HFpEF. Although, the survival rate of patients suffering from HF continues to drop, the management of HF still faces several limitations and discrepancies highlighting the need to develop new treatment strategies. Overstimulation of the sympathetic nervous system is an adaptive neurohormonal response to acute myocardial injury and heart damage, whereas prolonged exposure to catecholamines causes defects in βAR regulation, including a reduction in the amount of βARs and an increase in βAR desensitization due to the upregulation of G protein-coupled receptor kinases (GRKs) in the heart, contributing in turn to the progression of HF. Several studies show that myocardial GRK2 activity and expression are raised in the failing heart. Furthermore, β-arrestins play a pivotal role in βAR desensitization and, interestingly, can mediate their own signal transduction without any G protein-dependent pathway involved. In this review, we provide new insight into the role of GRKs and β-arrestins on how they affect βAR signaling regarding the molecular and cellular pathophysiology of HF. Additionally, we discuss the therapeutic potential of targeting GRKs and β-arrestins for the treatment of HF.

## Heart Failure

Heart failure (HF) is a heart disease with high morbidity and mortality. Based on measurement of the left ventricular ejection fraction (LVEF), HF with an LVEF less than 40% corresponds to HF with reduced ejection fraction (HFrEF) whereas HF with normal LVEF (≥50%) is termed HF with preserved ejection fraction (HFpEF). The therapeutic goals in patients with HF are to improve the clinical outcome and quality of life of HF patients, and also to reduce hospitalization and mortality rates. Angiotensin-converting enzyme inhibitors (ACEIs) and β-adrenergic receptor antagonists (β-blockers) have been shown to improve clinical outcomes and survival in patients with HFrEF and, therefore, are recommended for HFrEF treatment according to the ESC 2016 guideline for treatment of acute and chronic HF (Ponikowski et al., 2016). In several clinical trials, ACEIs, angiotensin receptor blockers (ARBs), mineralocorticoid receptor antagonists (MRAs), and β-blockers have been shown to reduce mortality and morbidity in patients with HFrEF (Ponikowski et al., 2016). However, none of these drugs have convincingly improved clinical outcomes and reduced morbidity/mortality in patients with HFpEF (Andersen and Borlaug, 2014; Ponikowski et al., 2016; Yamamoto, 2017; Bonsu et al., 2018).

Although β-blockers have dramatically reduced morbidity and mortality rates, β-blockers have limited effectiveness in some HF patients and have adverse effects. Thus, several barriers remain in the management of HF and new treatment strategies for HF need to be developed. In this review, we provide insight into the potential therapeutic targets for the treatment of HF, focusing in particular on G protein-coupled receptor kinases (GRKs) and β-arrestins.

## G Protein-Coupled Receptor Kinase

Upon agonist binding to β-adrenergic receptor (βAR), the heterotrimeric G proteins dissociate into Gα and Gβγ subunits which activate diverse downstream effectors and play fundamental roles in numerous cellular functions. Stimulation with an agonist simultaneously triggers the termination of the βAR signaling and the rapid reduction of the receptor responsiveness through a process called “receptor desensitization.” The phosphorylation by GRKs of agonist-occupied βAR corresponds to the first step of desensitization occurring within seconds to minutes which induces the recruitment of cytosolic β-arrestins to the receptor complex located on the plasma membrane. After binding, β-arrestins sterically inhibit further the interaction of βAR with Gαs resulting in their uncoupling of βAR. GRKs and β-arrestins also play an important role in βAR internalization, trafficking, and resensitization (Moore et al., 2007). In addition, GRKs can directly interact as scaffolding proteins with many signaling proteins, resulting in modulation of various physiological responses, and GRKs also phosphorylate several proteins other than receptors (Kurose, 2011; Watari et al., 2014).

GRKs preferentially phosphorylate GPCRs in an activated (agonist-bound) state at serine and threonine residues localized within either the third intracellular loop (ICL3) or C-terminal tail (Ferguson, 2001). This GRK-mediated phosphorylation of GPCR at these residues may regulate the stability of β-arrestin/GPCR complexes (Oakley et al., 1999). Even though GRK phosphorylation sites have been identified for some receptors, no distinct GRK phosphorylation consensus sequence/motif has been identified. The β_2_AR has a short ICL3 and a long C-terminal tail containing several serine and threonine residues. Mutation of all phosphorylation sites within the ICL and the C-terminal tail of β_2_AR attenuates GRK-mediated phosphorylation of receptors (Bouvier et al., 1988). In addition, the ICL3 of β_2_AR is associated with G protein activation and the specificity of the interaction between receptor and G protein (Reiter and Lefkowitz, 2006). On the contrary, human β_1_AR is resistant to GRK-mediated desensitization and internalization. Human β_1_AR does not internalize upon agonist stimulation and has lower affinity for β-arrestins than β_2_AR (Suzuki et al., 1992; Shiina et al., 2000). However, mouse β_1_AR is internalized by agonist stimulation (Volovyk et al., 2006). The physiological meaning of this species difference is unknown. Among GRKs, GRK2 has a fairly strict dependency of agonist binding for receptor phosphorylation, while GRK5 has the higher ability for phosphorylating agonist-unbound receptor as compared to GRK2 (Tran et al., 2007). GRK5-promoted phosphorylation of agonist-unbound receptor may help the receptor to activate β-arrestin-biased signaling that is primarily activated by antagonists. GRK-catalyzed phosphorylation of β_1_AR enhances β-arrestin-mediated signaling. It has been reported that β_1_AR-mediated biased signaling in the heart requires GRK5-promoted phosphorylation (Nakaya et al., 2012).

Interaction between GRKs and the activated βARs on the plasma membrane is necessary for GRK-catalyzed receptor phosphorylation. A recent study revealed a dynamic mechanism of complex formation between GRK5 and β_2_AR (Komolov et al., 2017). Two major domains of GRK5 [the regulator of G protein signaling homology (RH) and the catalytic domain] are able to dissociate following binding to activated β_2_AR causing disruption of a transient electrostatic contact between these two domains. These changes facilitate contacts between ICL2, ICL3, and the C-terminal tail of β_2_AR with the GRK5 RH bundle domain, the membrane-binding surface, and the kinase catalytic pocket, respectively (Komolov et al., 2017).

## GRK Family

According to their amino acids sequence and ternary structural homology, GRK family members can be divided into three groups as follows: rhodopsin kinase subfamily (GRK1 and GRK7), βAR kinase (βARK) subfamily (GRK2 and GRK3), and GRK4 subfamily (GRK4, GRK5, and GRK6) (Penn et al., 2000; Penela et al., 2003). The structure of GRKs consists of three distinct domains: an amino terminal (N-terminal) domain, a central highly conserved catalytic domain, and a carboxyl terminal (C-terminal) domain. The N-terminal domain involved in receptor binding and recognition of the activated receptor contains a region of homology to the regulator of G protein signaling protein and Ca^2+^/calmodulin-binding domain (Penn et al., 2000; Penela et al., 2003). The N-terminal domain of GRK4, GRK5, and GRK6 contains a phosphatidylinositol 4,5-bisphosphate (PIP_2_)-binding site allowing the amplification of their kinase activities (Pitcher et al., 1998), whereas the N-terminus of GRK2 contains a Gβγ-binding site causing GRK2 binding to the plasma membrane (Eichmann et al., 2003). The central domain is highly conserved among GRKs and exerts the kinase catalytic function.

The C-terminal domain of GRKs is involved in plasma membrane targeting and membrane binding by means of post-translational modifications or interaction with membrane phospholipids. The C-terminal domains of GRKs are divergent among subfamilies. GRK1 and GRK7 interact with the plasma membrane via a post-translational modification at their C-termini. The C-terminal domain of GRK2 and GRK3 is composed of a pleckstrin homology domain that includes a phospholipid-binding site and a Gβγ-binding site (Penn et al., 2000; Penela et al., 2003).

We reported in our previous study that GRK2 has a clathrin-binding motif at its C-terminus domain enabling the kinase to cooperate with the heavy chain of clathrin (Shiina et al., 2001), a step necessary for βAR internalization (Mangmool et al., 2006). GRK4 and GRK6 are post-translationally palmitoylated at cysteine residues located in their C-terminal domains, leading to plasma membrane localization and interaction. GRK5 binds to membrane phospholipids through electrostatic interaction of the positively charged amino acid located in its C-terminus. Finally, membrane localization of GRK4, GRK5, and GRK6 is regulated by the interaction with membrane PIP_2_ via their PIP_2_-binding sites (Penela et al., 2003). GRKs mediate the desensitization and internalization of βARs, including β_1_AR and β_2_AR. It is likely that subtype specificity of GRKs underlies observed differences in the regulation of βARs. In HEK-293 cells overexpressing GRK2, but not GRK5 and GRK6, the agonist-induced β_2_AR phosphorylation was inhibited by the treatment of clathrin heavy chain siRNA. These results suggest an important role for clathrin in GRK2-mediated β_2_AR phosphorylation and internalization (Mangmool et al., 2006). In addition, GRK2 phosphorylates four amino acid residues (Thr384, Ser396, Ser401, and Ser407) of the C-terminal tail of β_2_AR whereas GRK5 phosphorylates six amino acid residues (Thr384, Thr393, Ser396, Ser401, Ser407, and Ser411) of the C-terminal tail of β_2_AR (Fredericks et al., 1996).

More than hundreds of GPCRs have been identified in the human while seven members only of GRK family have been identified. It is not well understood why a limited number of GRKs regulate various GPCRs. The distribution pattern and expression levels of each GRK seem important factors contributing to their specificity and functional roles in many tissues, including the heart.

## GRK Expression in the Heart

GRK2 (known as βARK1), GRK3, and GRK5 are highly expressed in the human heart, whereas GRK4, GRK6, and GRK7 are only expressed at minimal levels (Ungerer et al., 1993). The distribution of each GRK isoform is different among heart cells. GRK2 and GRK5 are expressed in almost all cardiac cells, whereas GRK3 is detected only in cardiac myocytes (Vinge et al., 2001; Penela et al., 2006). The functional role of GRK subtypes in the heart under normal and pathological conditions may be influenced by their distribution. The functional role of GRK2 in cardiac fibroblasts was recently identified and GRK2 modulates contractility and remodeling following ischemia/reperfusion injury (Woodall et al., 2016). GRK expression and activity are changed in many cardiovascular diseases, especially HF. Thus, the functional roles of GRKs have been extensively studied in the heart as diagnostic markers and/or therapeutic targets for HF (Hullmann et al., 2016).

## Role of GRK in HF

Dysregulation of βARs is a pathological hallmark of HF; in particular, βARs are significantly downregulated and desensitized because of the upregulation of GRKs, especially GRK2 and GRK5 (Sato et al., 2015). βARs are targets for GRK-mediated phosphorylation and desensitization, and increased expression and activity of GRK2 in the heart are associated with the loss of βAR functions that induces deleterious effects. Although these events lead to the development and progression of HF, the inhibition of GRK2 expression or activity is able to restore cardiac functions (Petrofski and Koch, 2003).

In HF, the increase in catecholamine levels is derived from chronic sympathetic activation, resulting in overstimulation of βARs. The heart adapts to excessive stimulation by blunting the βAR responsiveness to catecholamines (Lymperopoulos et al., 2013; de Lucia et al., 2018). This process called βAR desensitization requires GRKs. However, desensitization and downregulation of βARs are not sufficient to compensate fully for chronic overstimulation of the sympathetic system. Prolongation of excessive stimulation over time becomes harmful to the heart and is responsible for the majority of HF (Lymperopoulos et al., 2013; de Lucia et al., 2018). Indeed, βAR desensitization is involved in the decrease of β_1_AR expression (βAR downregulation) and the uncoupling of βAR from G protein occurs during HF.

Because GRKs are involved in desensitization and downregulation of βAR, whether expression and activity of one or more GRKs is increased in HF patients or HF model animals has been extensively investigated in the past several years (Table 1). Chronic administration of isoproterenol resulted in βAR desensitization, upregulation of GRK2, and hypertrophy in mouse hearts (Iaccarino et al., 1998b). In addition, several studies have demonstrated that expression and activity of cardiac GRK2 are significantly increased in the failing heart (Ungerer et al., 1993; Harris et al., 2001; Rengo et al., 2011; Sato et al., 2015), indicating that upregulation of GRK2 plays a pivotal role in the HF associated with the dysfunction of βAR-mediated signaling.

**Table 1:** Changes in GRKs and β-arrestins levels and activities in animal models of HF and HF patients.

Experiments/Populations	Results	Reference
Human dilated cardiomyopathy	Increased GRK2 and GRK5 mRNA levels Unchanged GRK3 mRNA level	Dzimiri et al., 2004
Human failing heart	Elevated GRK2 mRNA level and activity in failing heart	Ungerer et al., 1993
Human failing heart	Increased GRK2 and GRK5 (but not GRK3) protein levels in left ventricles	Agüero et al., 2012
Human failing heart	Increased GRK2 mRNA level Slightly increased GRK3 mRNA level Unchanged β-arrestin1 and β-arrestin2 mRNA levels	Ungerer et al., 1994
Rabbit failing heart	Elevated GRK2 protein level and activity in post-myocardial infarction (post-MI) heart	Maurice et al., 1999
Isolated perfused rat heart	Increased GRK2 mRNA level and activity during myocardial ischemia	Ungerer et al., 1996
Rat model of congestive heart failure (CHF)	Increased GRK2, GRK5, β-arrestin1, and β-arrestin2 mRNA levels in failing heart Increased GRK2, GRK5, and β-arrestin1 in post-infarction failing heart	Vinge et al., 2001
Pacing-induced CHF in pig	Increased total GRK activity Increased GRK5 mRNA and protein levels Unchanged GRK2 mRNA and protein levels	Ping et al., 1997
Pressure-overload cardiac hypertrophy in mice	Increased GRK activity	Choi et al., 1997

Transgenic mice with cardiac-specific overexpression of GRK2 had contractile responses to βAR abolished and displayed physiological alterations (e.g., impairment of βAR functions and signaling, and cardiac hypertrophy), resulting in a failing heart in these mice (Koch et al., 1995; Rockman et al., 1998). Moreover, GRK2 high expression is detected in patients with end-stage dilated HF (Ungerer et al., 1994) and in several conditions related to HF development, including myocardial ischemia (Ungerer et al., 1996) and hypertension (Gros et al., 1997). Taken together, these results show that GRK2 dysfunction plays a pivotal role in heart diseases. However, the exact mechanism responsible for upregulation of GRK2 following βAR overstimulation in the compromised heart is not clearly understood.

Conversely, attenuation of GRK2 activity by expression of the carboxyl terminal domain of GRK2 (βARKct), which inhibits agonist-dependent GRK2 translocation to the membrane, or a reduction in GRK2 expression, enhanced cardiac functions (Koch et al., 1995). In addition, overexpression of βARKct could restore the diminished βAR contractile function and largely reverse the impaired cardiac functions in animal models of HF, such as muscle LIM protein (MLP) knockout (KO) mice (Rockman et al., 1998) and calsequestrin-overexpression mice (Harding et al., 2001). Thus, GRK2 expression in the heart appears to be related with cardiac contractile function. Previous studies have provided strong evidence that GRK2 plays a pivotal role in the βAR-mediated development of HF (Iaccarino et al., 1998b; White et al., 2000; Raake et al., 2008) and the increase of GRK2 expression can be used as an early marker for HF (Rengo et al., 2011; Lymperopoulos et al., 2013). Taken together, GRK2 acts as a central modulator of βAR signaling in the heart and could serve as a HF diagnosis biomarker.

Moreover, GRK2 also plays an important role in the βAR-mediated cardiac insulin resistance. Overstimulation of myocardial β_2_ARs and upregulation of GRK2 are associated with insulin resistance in the heart (Mangmool et al., 2017). Chronic stimulation of β_2_ARs, but not β_1_ARs, resulted in impaired insulin-induced glucose uptake and IRS-1 phosphorylation (Cipolletta et al., 2009) and also significantly reduced the actions of insulin to induce GLUT4 expression and translocation in cardiac myocytes and heart tissues (Mangmool et al., 2016). In addition, upregulation of β_2_AR enhances GRK2 expression that is related with βAR-induced insulin resistance in heart tissue (Ciccarelli et al., 2011) and in animal models of insulin resistance (Cipolletta et al., 2009). Thus, inhibition of GRK2 activity leads to enhanced insulin sensitivity in the heart. In animal models of diabetes, inhibition of GRK2 and GRK3 through synthetic peptides rescues glucose tolerance and improves insulin sensitivity (Anis et al., 2004). Other myocardial GRK isoform functions in this context, including GRK5, and the precise mechanism of GRK2 for βAR-mediated cardiac insulin resistance represent interesting areas of future research.

During pathological states of heart conditions, the expression of other GRK isoforms is also altered. GRK3 and GRK5 have been demonstrated to participate in the pathogenesis of HF. GRK5 expression is increased in animal models of HF (Ping et al., 1997; Vinge et al., 2001; Yi et al., 2002). GRK5 expression is also increased in dilated cardiomyopathy and volume-overloaded human left ventricle (Dzimiri et al., 2004), whereas the expression of GRK3 remains stable in dilated cardiomyopathy and is slightly induced in patients with right ventricular volume overload (Ungerer et al., 1993, 1996; Dzimiri et al., 2004). Nevertheless, dynamic alterations of GRK3 and GRK5 related to the development of HF remain to be elucidated.

In a model of transgenic mice overexpressing cardiac-specific GRK5, a considerable decline of βAR signaling and inotropic responsiveness was detected (Rockman et al., 1996). Interestingly, overexpression of GRK3 in mice does not affect βAR signaling and cardiac function (Iaccarino et al., 1998a), indicating that GRK3 does not share functional characteristics of GRK2, even though these two GRKs belong to the same subfamily. GRK3 might play a role desensitization of in α_1_AR rather than βAR. Phosphorylation of α_1_AR by GRK3 contributed to cardiac hypertrophy and dysfunctions that occur during chronic pressure overload (von Leuder et al., 2012). In addition, inhibition of GRK3 activity using GRK3ct preserves cardiac function and prevents the development of HF after chronic pressure overload (von Leuder et al., 2012).

Although overexpression of GRK5 in the heart results in attenuation of βAR-mediated signaling and function (Rockman et al., 1996), the complete deletion of GRK5 as in GRK5-KO mice does not significantly affect βAR-mediated responses in the heart (Gainetdinov et al., 1999). However, cardiac hypertrophy and failing heart after pressure overload are detected in transgenic mice with cardiac-specific overexpression of GRK5 (Martini et al., 2008). These detrimental effects of GRK5 are derived from its activity in the nucleus. GRK5 activity in the nucleus may be associated with a progression of a maladaptive cardiac hypertrophy that is independent of βARs (Martini et al., 2008). In addition, cardiac-specific deletion of GRK5 exhibits cardioprotective effects against pathological hypertrophy and HF after pressure overload (Gold et al., 2012). GRK5 is also found to be required for β_1_AR-mediated transactivation of epidermal growth factor receptor (EGFR) that confers cardioprotection in mice (Noma et al., 2007). Since GRK5 has been shown to have both detrimental and cardioprotective effects, regulation and compartmentalization of GRK5 in normal and failing hearts represent the most important issues when considering the inhibition of GRK5 as therapeutic target for HF.

The GRK5-Leu41 polymorphism of GRK5, with leucine at position 41 substituted for glutamine, is abundantly found in African American populations. Interestingly, GRK5-Leu41 is a gain-of-function genetic polymorphism that enhances desensitization of βAR (Liggett et al., 2008). GRK5-Leu41 allele decreases the activity of βAR signaling in a similar way to a partial blockade of βAR by β-blockers, promoting cardioprotective effects against experimental catecholamine-induced cardiomyopathy. HF patients with the GRK5-Leu41 allele show improved survival (Liggett et al., 2008), suggesting that modulation of GRK5 remains a powerful target for the treatment of HF. However, the specific contribution of each GRK isoform to the development of a failing heart remains to be determined.

## Therapeutic Approaches of GRKs for the Treatment of HF

Even though β-blockers inhibit HF progression and improve the quality of life in HF patients, these drugs show modest effectiveness in improving the contractile functions of the failing heart in animal models. If increased GRK activity and expression are important elements in desensitization and dysregulation of βAR in HF patients, potential therapeutic strategies aimed to modulate GRKs by preventing their expression and activity would consequently boost the ability of cardiac myocytes to respond to adrenergic stimulation (Koch et al., 1995; Korzick et al., 1997). For example, in animal models of HF, expression of βARKct, a GRK2 inhibitor, delays the progression of functional and biochemical modifications of the βAR signaling associated with HF (Rockman et al., 1998). Thus, inhibition of GRK by different strategies might be a novel therapeutic approach for restoring cardiac functions in the failing heart (Table 2). We will summarize results and strategies to inhibit GRK activity that could help to design novel therapeutic strategies for HF management.

**Table 2:** GRKs as the therapeutic targets for HF treatment.

Experiments/Populations	Results	Reference
Transgenic mice with cardiac-specific overexpression of βARKct	Overexpression of βARKct enhanced cardiac contractility and improved cardiac functions	Koch et al., 1995
Cardiac-specific overexpression of βARKct in HF model mice (MLP KO mice)	Overexpression of βARKct prevented the progression of cardiomyopathy	Rockman et al., 1998
Cardiac-specific overexpression of βARKct in HF model mice (calsequestrin overexpressed mice)	Overexpression of βARKct markedly prolonged survival and restored cardiac functions in failing heart	Harding et al., 2001
βARKct was expressed by adenovirus-mediated gene transfer in ventricular myocytes isolated from human failing heart	Expression of βARKct improved contractile function and βAR-mediated responses in failing human cardiac myocytes	Williams et al., 2004
GRK2 gene ablation in mice of post-MI model	Deletion of GRK2 before coronary artery ligation delayed maladaptive post-infarction remodeling and restored βAR signaling and functions GRK2 deletion initiated 10 days after MI enhanced survival, improved contractility, and inhibited cardiac remodeling	Raake et al., 2008
Mice of post-MI HF model	Paroxetine prevented HF development due to inhibition of GRK2 activity	Schumacher et al., 2015
Cardiac myocytes (*in vitro*) and mice (*in vivo*)	Paroxetine increased βAR-mediated cardiomyocyte contractility *in vitro* Paroxetine improved βAR-mediated left ventricular inotropic reserve *in vivo*	Thal et al., 2012

## βARKct (or GRK2ct)

βARKct, a polypeptide of 194 amino acids, consists of the Gβγ binding domain of GRK2 (Hullmann et al., 2016). Inhibition of GRK2 using βARKct dramatically improves cardiac contractility in animal models of HF (Hata et al., 2004). The mechanism of action proposed for βARKct is to inhibit the activity of endogenous GRK2 by competing with the endogenous GRK2 for Gβγ-binding, thus, attenuating GRK2 membrane translocation and activation, resulting in a reduction of GRK2-mediated βAR desensitization (Koch et al., 1995). Providing inhibitory βARKct to several animal models of HF leads to the delay of cardiac dysfunction and increased survival (Table 2). Overexpression of βARKct improves cardiac functions, prevents cardiac remodeling and cardiac hypertrophy, and increases survival rates in several animal models of HF (White et al., 2000; Shah et al., 2001). Moreover, cardiac-specific βARKct overexpression prevents cardiac remodeling and development of HF in MLP KO mice, a model of dilated cardiomyopathy with elevated GRK2 levels in the heart, suggesting that inhibition of GRK2 activity represents an approach to prevent the development of HF (Rockman et al., 1998). Similarly, cardiac-specific overexpression of βARKct results in cardiac contractility improvement, a delay of adverse remodeling, and a prolonged lifespan in HF model mice with calsequestrin overexpression, which show severe cardiomyopathy and markedly reduced survival rate (Harding et al., 2001). The beneficial effects of βARKct were enhanced by co-treatment with metoprolol, suggesting that inhibition of GRK2 provides a better clinical outcome than in HF patients treated only by β-blockers (Harding et al., 2001). When cardiac myocytes isolated from heart tissues from HF patients were infected by adenovirus expressing βARKct, the βARKct-overexpressing myocytes exhibited significant increases of the heart ability to contract and relax in response to adrenergic stimulation (Williams et al., 2004). Thus, inhibition of GRK2 in the failing heart has beneficial effects on cardiac performance. However, it should be noted that βARKct might mediate its beneficial effects via mechanisms distinct from inhibiting GRK2, as βARKct is able to inhibit several Gβγ-mediated signaling pathways.

Even though gene therapy using βARKct represents a promising strategy for the treatment of HF, the large size of βARKct, and the virus requirement for heart-specific expression may represent major obstacles for clinical development. Small peptide or synthetic compounds that specifically inhibit GRK activity (selective GRK inhibitor) may provide a much easier way at a lower cost when searching for therapeutic approaches for HF. These data support the use of GRK2 inhibitor, including βARKct as a promising therapeutic approach for the treatment of chronic HF (Rengo et al., 2011). The effects of GRK2 inhibition might be similar or greater than those of classical β-blocker therapy (Reinkober et al., 2012).

## Paroxetine

Paroxetine is a selective serotonin reuptake inhibitor (SSRI) antidepressant that is found to be a GRK2 inhibitor (Homan et al., 2014). Paroxetine binds to GRK2 and inhibits GRK2 catalytic activity more potently than other GRK isoforms (Thal et al., 2011). From crystallization and structure analysis, paroxetine was reported to occupy the active site of GRK2 leading to the stabilization of the GRK2 kinase domain in a unique inactive conformation (Thal et al., 2011). Paroxetine enhances βAR-mediated shortening and contraction of isolated cardiac myocytes and increases βAR-mediated left ventricular inotropic reserve *in vivo* (Thal et al., 2012), similar to βARKct.

In addition, administration of paroxetine to an HF mouse model demonstrated an improvement of LV structure and function and attenuated the expression of the fetal genes, representing an index of progression to HF (Schumacher et al., 2015). These cardioprotective effects of paroxetine are due to the inhibitory activity of GRK2 but not the SSRI activity of paroxetine, suggesting that paroxetine prevents HF progression in an SSRI-independent manner. Moreover, equivalent doses of fluoxetine, another SSRI, did not show any of these effects. The result emphasizes that the effects of paroxetine are due to GRK2 inhibition (Schumacher et al., 2015). Thus, paroxetine-mediated inhibition of GRK2 enhances cardiac performance, reverses sympathetic overstimulation, normalizes the myocardial βAR functions, and protects the heart after myocardial infarction (MI) (Table 2). These data demonstrate that paroxetine-mediated inhibition of GRK2 improves cardiac function after MI and represents a potential repurposing of this drug, as well as starting point for innovative small-molecule GRK2 inhibitor development.

Interestingly, chronic treatment with various SSRIs (e.g., fluoxetine, paroxetine) stimulated serotonin receptor type 4 (5-HT_4_R) desensitization in cerebral regions implicated in depression, including basal ganglia and hippocampus (Licht et al., 2009; Vidal et al., 2009). Moreover, upregulation of GRK2 significantly suppressed serotonin-induced cAMP generation in COS-7 cells, suggesting a negative regulatory role for GRK2 at the 5-HT_4_R level (Barthet et al., 2005). In addition, serotonin-induced 5-HT_4A_R internalization was inhibited by expression of either dominant negative (DN) GRK2 and DN β-arrestin1 (βarr_1319-418_) in HEK-293 cells, suggesting that GRK2 and β-arrestin are involved in the trafficking of 5-HT_4_Rs (Mnie-Filali et al., 2010).

GRK-mediated phosphorylation of 5-HT_4_R exhibits high affinity for β-arrestins, which binds to the phosphorylated receptor and inhibit coupling with G proteins. This results in the inhibition of further signaling (Mnie-Filali et al., 2010). The 5-HT_4_R/GRK/β-arrestin complex may confer different signaling and regulatory characteristics to the 5-HT_4_ receptors, leading perhaps to new functional roles and eventually therapeutic implications. Since paroxetine is an inhibitor of GRK2 that plays an important role in 5-HT_4_R desensitization and trafficking, the exact mechanisms by which paroxetine affects the desensitization and the trafficking of various serotonin receptors, especially 5-HT_4_R remain to be elucidated.

## Other Synthetic GRK Inhibitors

Since GRK2 and GRK5 are upregulated in the failing heart and play major roles in the progression of cardiac dysfunction, including HF, a selective inhibitor of GRK2 and/or GRK5 might represent a promising target for HF treatment. Balanol is a synthetic compound that was found to inhibit GRK2 activity (Setyawan et al., 1999; Tesmer et al., 2010). However, balanol also inhibits other protein kinases such as protein kinase A (PKA), protein kinase C (PKC), and protein kinase G (PKG). A selective GRK2 inhibitor was developed by a two-step rational drug design process, and compound 10 (Methyl 5-[2-(5-nitro-2-furyl)vinyl]-2-furoate) was found to inhibit GRK2 more selectively than PKA (Iino et al., 2002). This compound was the first inhibitor that was able to distinguish GRK2 from PKA, a protein kinase that has a similar adenine-binding pocket.

A GRK2 inhibitor screening has been performed using a compound library, leading to identify various novel candidates (Homan et al., 2015). Currently, most active compounds can be divided into two chemical classes: indazole/dihydropyrimidine-containing compounds that strongly inhibit GRK2 and pyrrolopyrimidine-containing compounds that are selective for inhibition of GRK1 and GRK5 (Homan et al., 2014, 2015). Several new GRK inhibitors are also synthesized such as GSK180736A and GSK2163632A. These compounds were co-crystallized with GRKs (Homan et al., 2015). GSK180736A is a selective GRK2 inhibitor, which binds to GRK2 in a similar way to paroxetine, whereas GSK2163632A occupies a novel region of the GRK active site that is related to its selectivity (Homan et al., 2015). Further development by in silico screening, using GSK180736A and CCG215022 as templates, identified two new compounds (compounds 33 and 37) as potent GRK2 and GRK5 inhibitors (Waldschmidt et al., 2018). The IC_50_ value of GSK180736A toward GRK2 and GRK5 are 0.77 μM and >100 μM, respectively (Waldschmidt et al., 2018). However, the screening did not identify any compounds that exhibited high GRK5 selectivity.

Overstimulation of βAR is associated with the pathogenesis of insulin resistance in the heart (Mangmool et al., 2017). For instance, chronic βAR stimulation causes the development of insulin resistance through an increase in GRK2 levels (Cipolletta et al., 2009). Moreover, upregulation of GRK2 impairs cardiac glucose uptake and promotes insulin resistance after MI (Ciccarelli et al., 2011). The small peptides (e.g., KRX-683107 and KRK-683124) derived from the catalytic domain of GRK2 and GRK3 have been shown to improve glucose metabolism. By modulating GRK2 and GRK3 activities, these two peptides enhanced GPCR-mediated signal transduction, resulting in an antidiabetic effect (Anis et al., 2004). In animal models of diabetes, inhibition of GRK2 and GRK3 through these synthetic peptides rescues glucose tolerance and enhances insulin sensitivity (Anis et al., 2004). Thus, these small peptides could be useful as GRK inhibitors by interfering with kinase-substrate interactions. However, the efficacies of these small peptides in animal model of HF are not known. To develop specific inhibitors for GRKs, the sequence of the first intracellular loop (ICL1) of the β_2_AR was synthesized and found to inhibit GRKs (Winstel et al., 2005). IC_50_ value of a peptide with the sequence AKFERLQTVTNYFITSE for GRK2 is 0.6 μM. This peptide also inhibited GRK3 and GRK5 with an IC_50_ of 2.6 and 1.6 μM, respectively (Winstel et al., 2005). Furthermore, the peptide inhibitor did not suppress PKC and PKA activities because ICL1 of β_2_AR presents selectivity GRK2 over PKC and PKA (Benovic et al., 1990). However, the efficacy of these synthetic and peptide inhibitors of GRKs remain to be tested in animal models of HF, which means that another 6 to 10 years are needed before they can be tested in human if they show promising effects in animal models and preliminary safety data are obtained.

## β-Arrestins

Stimulation of βARs with agonists leads to GRK-mediated receptor phosphorylation that promotes the recruitment of β-arrestins to phosphorylated βARs. Then β-arrestins sterically inhibit further G protein coupling to βARs. After β-arrestin binds to and forms a complex with βAR and GRK, β-arrestin promotes internalization of βAR into the cytosol, which can lead to receptor degradation (downregulation) and receptor recycling back to the plasma membrane (Moore et al., 2007). Thus, β-arrestins play essential roles in βAR internalization and trafficking (Lefkowitz and Shenoy, 2005; Lefkowitz et al., 2006).

Although both β_1_ARs and β_2_ARs are Gs protein-coupled receptors, their functional properties differ and lead to subtype-specificity interaction with β-arrestins. For instance, the binding of β-arrestin-1 and -2 to the third intracellular loop (ICL3) and the C-terminal tail of β_1_AR is lower than that for β_2_AR (Shiina et al., 2000). A chimeric β_2_AR containing the C-terminal tail of β_1_AR lost its ability to promote β-arrestin2-mediated ERK nuclear translocation (Kobayashi et al., 2005). Moreover, stimulation of β_1_AR, but not β_2_AR, induces a conformational change in β-arrestin that promotes a stable β-arrestin/Epac/Ca^2+^/calmodulin-dependent protein kinase II (CaMKII) complex (Mangmool et al., 2010). The association of β-arrestin with β_1_AR stabilizes this complex and promotes CaMKII signaling (Mangmool et al., 2010). It is interesting that β-arrestin deletion results in differential ERK activation in a βAR subtype-selective manner (O’Hayre et al., 2017; Grundmann et al., 2018). Deletion of β-arrestin results in enhancement of β_2_AR-mediated ERK activation, but decrease of β_1_AR-mediated ERK activation. Enhancement of β_2_AR-mediated ERK activation is due to the impaired desensitization of β_2_AR and decrease of β_1_AR-mediated ERK activation is due to the lack of the scaffolding function of β-arrestin.

In addition to regulate GPCR endocytosis and trafficking, β-arrestins themselves function as scaffolding proteins and signal transducers to stimulate various downstream effectors, including MAPK cascades induction (e.g., ERK1/2 and JNK3), Src activation (Lefkowitz and Shenoy, 2005; Lefkowitz et al., 2006), and EGFR transactivation (Noma et al., 2007). Furthermore, β-arrestins can interact and form a complex with CaMKII (Mangmool et al., 2010). Complex formation facilitates CaMKII activation, which plays a key role in cardiac hypertrophy and apoptosis that cause HF (Mollova et al., 2015). The ability of β-arrestins to desensitize βARs is well established. In the present paper, we will mainly review the roles of β-arrestins in βAR-mediated signaling in normal and HF conditions, their function in the heart, and their potential as therapeutic target for HF treatment.

## β-Arrestin Family and Structure

The arrestin family consists of four members. Arrestin1 and arrestin4 (known as visual arrestin, and cone arrestin, respectively) are expressed in the rods and cones of eyes, respectively. The β-arrestin1 and β-arrestin2 (known as arrestin2 and arrestin3, respectively) are abundantly expressed throughout mammalian tissues (Gurevich and Gurevich, 2004). Arrestin contains an N-domain and a C-domain, each consisting of seven stranded β-sheets, linked through a short linker region (Graznin et al., 1998; Hirsch et al., 1999).

The N-domain of arrestins contains a recognition region for activated receptors and the C-domain contains a secondary receptor recognition region (Gurevich et al., 1995). The phosphate sensor region is located in the linker between N-domain and C-domain and forms part of the hydrophilic core of arrestins. The interaction between the end of C-domain and the phosphate sensor region maintains the arrestin structure in the resting state. In the active state, this interaction is disrupted upon receptor binding, allowing arrestin to bind the phosphorylated receptor with a high affinity (Gurevich and Gurevich, 2004).

## Role of β-Arrestin in βAR Desensitization

Agonist binding to βAR promotes receptor coupling with heterotrimeric G proteins and triggers the dissociation into activated Gα_s_ and the Gβγ subunits, which stimulates adenylyl cyclase (AC) and increases cAMP levels. cAMP binds to and interacts with its downstream effectors, resulting in activation of cAMP signal transduction. Subsequent to agonist binding, activated βARs are phosphorylated by GRKs leading to recruitment of β-arrestins and inhibition of further interaction of receptor with G proteins. This process is known as “receptor desensitization” as described previously (Ferguson, 2001; Luttrell and Lefkowitz, 2002).

## Role of β-Arrestin in βAR Trafficking

In addition to their regulation of βAR desensitization, β-arrestins are essential for βAR trafficking to intracellular compartments located in the cytosol. This process is called “βAR internalization.” Currently, β-arrestins are reported to interact with regulatory proteins required for receptor internalization, including clathrin, AP-2, guanine-nucleotide exchange factors, phosphoinositides, and GTPase activating proteins (reviewed in DeWire et al., 2007). After their internalization, βARs are directed toward two main intracellular pathways either degradation or recycling (Tan et al., 2004). The βARs targeted for recycling are transported to early endosomes where the receptors are dephosphorylated by protein phosphatase 2A (PP2A) (known as βAR dephosphorylation) before being transported back to the plasma membrane. Dephosphorylation of βARs is dependent on the acidification in early endosomes because acidification enhances PP2A catalytic function (Krueger et al., 1997). The βARs targeted for degradation are transferred to lysosomes where they are eventually degraded via ubiquitination mediated degradation (Tan et al., 2004). Both GRK-mediated receptor phosphorylation and β-arrestin binding to β_2_ARs are necessary for β_2_AR ubiquitination (Shenoy et al., 2001). However, the role of β-arrestin mediated βARs targeting for recycling and degradation is poorly understood.

## β-Arrestin Acts as Scaffolding Proteins

In addition to their roles in receptor desensitization and internalization, β-arrestins are recognized as multifunctional scaffolding proteins that work as adaptor proteins linking receptors to several downstream effectors such as ERK1/2 (Luttrell et al., 2001; Shenoy et al., 2006), JNK (McDonald et al., 2000), Src (Luttrell et al., 1999), and calmodulin (Wu et al., 2006). β-Arrestins are also known to bind to and activate CaMKII following β_1_AR stimulation (Mangmool et al., 2010). Interestingly, proteomic analysis by mass spectrometry demonstrated that β-arrestins bind to and interact with various types of proteins that play roles in cellular signaling (Xiao et al., 2007). Their abilities to form multifunctional protein complexes are related to functionally selective responses. β-arrestins play an essential role in the CaMKII signaling responsible for cardiac hypertrophy and HF. Stimulation of β_1_AR promotes a conformational change in β-arrestin which then induces the formation of a stable complex, including β-arrestin, CaMKII, and cAMP-dependent guanine-nucleotide exchange factor (Epac) (Mangmool et al., 2010). The role of β-arrestin in this multiprotein complex consists of holding Epac and CaMKII in structural proximity to activate CaMKII signaling (Mangmool et al., 2010). Discovering the mechanism of multifunctional complex formation by β-arrestins will help to understand the physiological importance of this scaffolding protein complex in HF.

## Role of β-Arrestins in the Failing Heart

Downregulation and desensitization of βARs occur in the failing heart, leading to dramatically diminish cardiac functions via reduced contractility (Brodde, 1993; Port and Bristow, 2001). The modulation mechanism of βARs is elucidated at the molecular level and involves GRKs and β-arrestins. Two isoforms of β-arrestin are expressed in the heart, namely β-arrestin1 and β-arrestin2 (Ungerer et al., 1994). The signaling mediated by β-arrestins independently to classical G protein-mediated signaling may be associated with cardioprotective effects (Patel et al., 2009; Noor et al., 2011). However, the specific roles of each β-arrestin isoform in cardiac βAR dysfunction, leading to the pathophysiology of HF are not fully understood.

## Cardioprotective Effects of β-Arrestins

McCrink et al. (2017) have demonstrated that overexpression of β-arrestin2 in mice restores inotropic reserves of β-adrenergic regulation. They have shown that β-arrestin2 directly binds to and activates sarcoplasmic/endoplasmic reticulum Ca^2+^-ATPase 2a (SERCA2a), a key regulator of β_1_AR-dependent cardiac contractility. The association of β-arrestin2 with SERCA2a contributes to SERCA2a SUMO (small ubiquitin-like modifier)-ylation and then increases SERCA2a activity, leading to increased cardiac contractility (McCrink et al., 2017). In contrast, β-arrestin1 had no effect on SERCA2a activation.

Although β-arrestin2 expression is low in the mammalian heart, including humans (Ungerer et al., 1994), β-arrestin2 has cardioprotective roles against HF. Thus, cardiac-specific β-arrestin2 gene transfer is a very attractive approach for gene therapy in HF patients. Another approach to treatment of HF is selective activation of β-arrestin2, although the expression level of β-arrestin2 is low in the heart. TRV120067 is a β-arrestin–biased ligand targeted to the angiotensin II type 1 receptor (AT_1_R) that works similar to ARBs for selective blockade of Ang II binding to AT_1_R and subsequent G protein coupling, while simultaneously and preferentially activates β-arrestin2-dependent signaling. TRV120067 exhibits cardioprotective effects in a mouse model with dilated cardiomyopathy (Ryba et al., 2017), suggesting that β-arrestin2-dependent signaling confers significant benefits on cardiac function and structure. β-Arrestin2 constitutively localizes in cardiac sarcomeres, and the localization is enhanced by TRV120067 that activates β-arrestin2 and the downstream effector SERCA2a (Ryba et al., 2017).

Many studies have demonstrated that excessive inflammation induces detrimental effects on the heart after MI. Because inflammatory cytokines are reported to induce cardiomyocyte apoptosis (Ing et al., 1999; Li et al., 2007), it is possible that they enhance apoptosis in the infarct area. However, inflammation is also reported to be necessary for recovery of the heart from MI-induced injury. Monocyte subsets phagocytose dead cells and produce anti-inflammatory cytokines such as TGF-β (Frangogiannis, 2008). TGF-β plays a crucial role in cardiac repair by suppressing inflammation and promotes differentiation of fibroblasts into myofibroblasts that produce extracellular matrix. Although many reports on the roles of inflammation in MI, it has not been established whether inhibition or enhancement of inflammation is protective against MI-induced cardiac dysfunction and remodeling in clinical practice.

Among GRKs and β-arrestins, β-arrestin2 is an interesting target for the treatment of HF. β-Arrestin2 delays inflammatory responses by interfering with macrophage recruitment to the infarcted area. β-Arrestin2 is highly expressed in infiltrated macrophages, resulting in the inhibition of excessive inflammation and apoptosis after MI (Watari et al., 2013). The level of many inflammatory cytokines was higher in β-arrestin2 KO mice than in wild-type (WT) mice after MI, showing that β-arrestin2 has a protective role in inflammatory processes induced by MI. Moreover, the mortality rate of β-arrestin2 KO mice was increased (Watari et al., 2013). Furthermore, β-arrestin2 has been shown to prevent cell apoptosis (Ahn et al., 2009; Yang et al., 2012). These results indicate that β-arrestin2 in infiltrated macrophages plays an essential role in the inhibition of excessive inflammation after MI, and inhibition of β-arrestin2 function prevents myocyte apoptosis against ischemic injury. Several studies have shown the importance of β-arrestin2-mediated signal transduction in the heart; in particular, β-arrestin2 protects the heart against overstimulation of βAR in *in vitro* and *in vivo* studies (Noma et al., 2007). Therefore, pharmacological activation of β-arrestin2 might represent a beneficial therapy in HF.

It has been reported that β_1_AR (Noma et al., 2007) and β_2_AR (Shenoy et al., 2006) mediate ERK1/2 signaling in a β-arrestin-dependent manner. Stimulation of β_1_AR results in transactivation of EGFR, which activates ERK signaling (Noma et al., 2007). EGFR transactivation following βAR stimulation is mediated by a GRK-dependent and β-arrestin-dependent pathway, which exhibits cardioprotective effects under conditions of excessive catecholamine stimulation (Noma et al., 2007). These results also suggest that the effects of G protein-mediated signaling may contribute to the detrimental cardiac remodeling observed during β_1_AR overactivation.

A model of β_1_AR signaling in the heart has been proposed, in which β_1_AR mediates two distinct signaling pathways following receptor stimulation (Noma et al., 2007). The G protein-dependent signaling might be harmful and cause detrimental effects under excessive catecholamine stimulation, whereas β-arrestin-mediated signaling that is able to transactivate EGFR to evoke cardioprotective effects in response to the same pathological stimuli in the heart (Noma et al., 2007). Although EGFR activation results in cardiac hypertrophy, whether β-arrestin-mediated transactivation of EGFR is associated with cardiac hypertrophy is unknown.

## β-Arrestin1 Has Detrimental Effects to the Heart

The role of β-arrestin1 in remodeling of post-MI has been investigated by using β-arrestin1-knockout (-KO) mice and WT mice under normal conditions and after surgical MI operation (Bathgate-Siryk et al., 2014). Normal (sham-operated) β-arrestin1-KO mice display enhanced βAR-dependent contractility due to impairment of βAR desensitization. After MI, β-arrestin1-KO mice display enhanced overall cardiac function (and βAR-dependent contractility) compared to WT mice. β-Arrestin1-KO mice also show increased survival, and decreased cardiac infarct size, apoptosis, and adverse remodeling, as well as circulating catecholamines and aldosterone, compared to WT mice after MI. The underlying mechanisms are; (1) on one hand improved cardiac βAR signaling and function, as evidenced by increased βAR density and pro-contractile signaling, via reduced cardiac βAR desensitization due to cardiac β-arrestin1 absence, and (2) on the other hand decreased production leading to lower circulating levels of catecholamines and aldosterone due to adrenal β-arrestin1 absence. Thus, β-arrestin1, via both cardiac and adrenal effects, is detrimental for cardiac structure and function and significantly exacerbates development of HF after MI. Thus, β-arrestin1 might be an important negative regulator of βAR-mediated cardiac signaling and functions through the classical processes of βAR desensitization and downregulation. β-Arrestin1 may be a salient β-arrestin isoform that is responsible for βAR desensitization and downregulation in the heart, leading to progression of cardiac abnormality and dysfunction. In contrast, stimulation of β-arrestin2 through βAR leads to EGFR transactivation and ERK1/2 activation that promotes cell survival and proliferation. Thus, the actions of two isoforms of β-arrestin (β-arrestin1 and β-arrestin2) might counteract each other in certain cells and tissues, including in the heart (Rajagopal et al., 2006; Kim et al., 2008). It also suggests that inhibition of β-arrestin1 function in the heart by either a specific inhibitor or via genetic manipulation has beneficial effects on HF. However, the underlying mechanisms and actions of each β-arrestin in desensitization and downregulation of βAR, and in G protein-independent signaling remain to be clarified.

## β-Arrestin-Biased Ligands for βAR

The βAR antagonist is a ligand that binds to βAR but cannot activate the receptor. It also has the ability to antagonize agonist-stimulated βAR. However, the concept of agonist and antagonist is challenged by the findings that ligands for βARs could be an antagonist for the G protein-mediated signaling and also act as agonists for the β-arrestin-mediated signal transduction (Wisler et al., 2007; Kim et al., 2008; Wisler et al., 2014). When a ligand selectively activates one of the G protein-dependent and β-arrestin-dependent signaling pathway, the ligand is called as “biased ligand.” Thus, a β-arrestin-biased ligand is a ligand that can antagonize the receptor-mediated G protein activation and at the same time activate signaling pathways in a G protein-independent but β-arrestin-dependent manner (Violin and Lefkowitz, 2007). β-Arrestin-biased ligands are expected to have beneficial effects on HF because of their selective activation of β-arrestin signaling that mediates favorable physiological responses in the heart (Noor et al., 2011). Discovery of novel biased ligands for βARs that are able to block G protein-mediated signaling but stimulate β-arrestin-mediated signaling represents potential therapeutic treatment for HF.

A β-arrestin-biased ligand is believed to activate an alternative signaling pathway due to the stabilization of the receptor in a particular distinct conformation, resulting in the biased activation of G protein- or β-arrestin-dependent signaling. In contrast, unbiased ligand for βAR (e.g., isoprenaline) binds to and stabilizes the βAR conformation that equally activates G protein and β-arrestin (Rajagopal et al., 2010; Wisler et al., 2014; Bologna et al., 2017). We will mainly summarize here the beneficial effects of β-arrestin-biased ligands for βAR.

Long-term use of β-blockers clinically delays progression of HF by reducing cardiac remodeling and correcting left ventricular contractility in the failing heart (López-Sendón et al., 2004). β-Blockers may regulate the βAR system by modulating β_1_AR functions and reversing receptor sensitivity. Moreover, administration of β-blockers has been reported to increase βAR responsiveness and decrease GRK2 expression (Iaccarino et al., 1998b), contributing to the sensitization of βAR functions and signaling. Nevertheless, each β-blocker shows a unique effect on βAR-mediated signaling. β-Blockers differ in terms of βAR subtype selectivity, ability to block αAR, antioxidant activity, and anti-inflammatory activity (Barrese and Taglialatela, 2013). Some β-blockers are β-arrestin-biased ligands, including carvedilol, metoprolol, and nebivolol.

## Interaction of β-Arrestin-Biased Ligand With βAR

The binding of agonists and antagonists can evoke differential conformational changes of βARs. Biased ligands (or biased agonist) can induce distinct βAR conformations that selectively activate specific signaling pathways, different from full agonists and antagonists (Thanawala et al., 2014). β-Arrestin-biased ligands induce and stabilize a ligand-dependent unique receptor conformation and then selectively activate the particular signaling pathway (Thanawala et al., 2014; McCorvy et al., 2018). Stimulation of vasopressin type 2 receptor (V2R) by a G protein-biased ligand stabilizes V2R in a conformation different from that stabilized by a β-arrestin-biased ligand (Rahmeh et al., 2012). In particular, the third intracellular loop and transmembrane domain 6 (TM6) regions of V2R are necessary for G protein-mediated signaling, whereas the TM7 and putative helix 8 are required for β-arrestin-mediated signaling (Rahmeh et al., 2012). Thus, the functional outcome of biased ligands depends on which ligands stabilized conformation is favorable for G protein- or β-arrestin-coupling.

In addition, site-specific fluorine-19 nuclear magnetic resonance (19F-NMR) of β_2_AR has shown that ligand binding to β_2_AR modulates G protein- and β-arrestin-dependent signaling by inducing distinct conformations of the receptor depending on stimulation with either unbiased or biased ligands (Liu et al., 2012). Unbiased ligands bind to β_2_AR and induce the conformational change of helix 6 into the active state that specifically leads to activate G protein signaling. In contrast, β-arrestin-biased ligands predominantly induce the conformational change of helix 7 of β_2_AR that is necessary for β-arrestin-mediated signal transduction (Liu et al., 2012). Moreover, the tyrosine residue at position 308 (Tyr-308) of β_2_AR is found to be essential for Gs protein-biased signaling of β_2_AR. The unique interaction between ligand and Tyr-308 of TM7 in β_2_AR stabilizes the receptor conformation favoring the β_2_AR-Gs protein coupling that is necessary for G protein-dependent signaling (Woo et al., 2014).

Roth’s group has reported that ligand binding to amino acid residues at TM5 and extracellular loop 2 (ECL2) of the receptor are important for Gi/o protein and β-arrestin signaling, respectively (McCorvy et al., 2018). They targeted these residues to develop both G protein- and β-arrestin-biased ligands for aminergic GPCRs that contain similar residues at TM5 and ECL2, including βARs. Ligand contacts with TM5 of GPCR trigger the conformational change to induce a cytoplasmic inward movement of TM5 (Warne and Tate, 2013). This movement change then results in movement of ICL2 and TM6 regions that are involved in G protein coupling and activation (Deupi and Standfuss, 2011; Rasmussen et al., 2011). In contrast, the interaction of biased ligand with ECL2 of β_2_AR is key for β-arrestin recruitment. ECL2 is an important region that locks the ligand into the binding site, resulting in an increased period of ligand binding and promotion of β-arrestin recruitment required for β-arrestin-mediated signaling pathway. Thus, TM5 and ECL2 are the regions to be focused on to develop specific biased ligands (G protein-biased and β-arrestin-biased ligands) of βARs that possess desirable therapeutic effects with minimal adverse effects.

Lefkowitz’s group has used bioluminescence resonance energy transfer-based biosensor of β-arrestin2 to detect the β-arrestin conformational change upon biased ligand binding to receptor (Shukla et al., 2008). Their study showed that β-arrestin can convert βAR into multiple conformations and each unique β-arrestin-favorable conformation can form a complex with different binding proteins and evoke a corresponding specific signal transduction. Although these studies have demonstrated molecular mechanisms and interactions of biased ligand with specific regions of the receptor, further studies are required to establish the effects of β-arrestin-biased ligands in cellular functions in normal and pathophysiological conditions such as HF.

βARs can modulate the contractile function of the heart. Catecholamines, such as adrenaline and noradrenaline, activate cardiac β_1_AR and β_2_AR, which then activates the canonical Gs/AC/cAMP signaling cascade. Cyclic AMP binds to and activates its downstream effectors, including PKA (Salazar et al., 2007). PKA phosphorylates a set of regulatory proteins that are essential for cardiac contractility such as the voltage-gated L-type Ca^2+^ channel, the cardiac ryanodine receptor, phospholamban, and some myofilament components (troponin I and troponin C) (Salazar et al., 2007). PKA mediates phosphorylation and activation of L-type Ca^2+^ channel and ryanodine receptor results in significant increase in intracellular Ca^2+^ levels, which is necessary for cardiac muscle contraction. Furthermore, βAR-mediated phosphorylation of phospholamban (Sulakhe and Vo, 1995), a negative modulator of SERCA, accelerates Ca^2+^ reuptake into the sarcoplasmic reticulum (SR), and increases SR Ca^2+^ stores available for the next contraction (Brittsan and Kranias, 2000). In addition, troponin I phosphorylation by PKA reduces myofilament sensitivity to Ca^2+^ following βAR stimulation (Endoh and Blinks, 1988), hence inhibiting contractile signaling and accelerating cardiac relaxation (Zhang et al., 1995). Some β-blockers act as β-arrestin-biased ligands that can inhibit classical Gs protein signaling and stimulate β-arrestin signaling. The ability to activate biased signaling may explain the clinical differences between treatment with classical β-blockers and β-arrestin-biased β-blockers. We summarize below recent advances on β-arrestin-biased β-blockers that are used in clinic for the treatment of HF as presented in Table 3.

**Table 3:** Effects of β-arrestin-biased β-blockers.

β-Arrestin-biased β-blockers	Experiments/Models	Effects	Reference
Alprenolol and Carvedilol	*In vitro* (using HEK-293 cells) and *in vivo* (using mice) studies	Alprenolol and carvedilol stimulated β-arrestin-mediated EGFR transactivation and ERK1/2 activation	Kim et al., 2008
Carvedilol and Propranolol	Rat hippocampal neurons	Carvedilol and propranolol that inhibit βAR signaling via G proteins, mediated neuronal calcium signaling through β-arrestin2 and ERK1/2	Tzingounis et al., 2010
Carvedilol	β_2_AR-expressing HEK-293 cells	Carvedilol stimulated β-arrestin-dependent ERK1/2 activity in absence of G protein activation	Wisler et al., 2007
Metoprolol	*In vitro* (using cardiac myocytes) and *in vivo* (using GRK5-KO and β-arrestin2-KO mice) studies	Metoprolol caused cardiac fibrosis in a G protein-independent and GRK5/β-arrestin2-dependent manner	Nakaya et al., 2012
Nebivolol	*In vitro* (using mouse embryonic fibroblasts and cardiac myocytes)	Nebivolol-mediated ERK1/2 activation was inhibited by inhibition of GRK2 as well as knockdown of β-arrestin1/2	Erickson et al., 2013

## Carvedilol

Carvedilol is a nonselective β-blocker that can antagonize both β_1_- and β_2_-AR. Blockade of βARs in the heart by carvedilol improves cardiac function, including contractility, and attenuates myocardial remodeling in the failing heart (Iaccarino et al., 1998b; Kukin et al., 1999). In addition to nonselective blockade of βAR, carvedilol has other characteristics, including α_1_-adrenergic blockade, antioxidant, anti-proliferative, anti-inflammatory, and vasodilating effects, which may explain why its efficacy is higher than other β-blockers (Metra et al., 2005; Pedersen and Cockcroft, 2007; Barrese and Taglialatela, 2013). Interestingly, carvedilol has been classified as a β-arrestin-biased ligand for βARs (Wisler et al., 2007; Kim et al., 2008).

Among the 16 available β-blockers, carvedilol is the only β-blocker that can activate ERK signaling pathway by a β_2_AR-mediated, G protein-independent, and β-arrestin-dependent mechanism (Wisler et al., 2007). Alprenolol and carvedilol can activate the β_1_AR-stimulated transactivation of EGFR through β-arrestin-mediated signaling without activation of G proteins (Kim et al., 2008). Therefore, carvedilol is different from other β-blockers, as it acts as a β-arrestin-biased ligand that exhibits cardioprotective effects in *in vitro* and *in vivo* studies (Table 3). Carvedilol-mediated β-arrestin-biased signaling might contribute to its clinical profile.

On the basis of studies of receptor structure, carvedilol can stabilize divergent receptor conformations and induce phosphorylation of βAR. The phosphorylation sites of βAR by carvedilol are different from those of unbiased ligands (Nobles et al., 2011; Liu et al., 2012). In a meta-analysis, carvedilol has been reported to have superior beneficial effects compared with other β_1_-selective β-blockers (i.e., atenolol and bisoprolol) in post-MI (DiNicolantonio et al., 2013). Carvedilol has more potent effects on the reduction of mortality and morbidity in acute MI and HF compared with other β-blockers in randomized comparison trials (DiNicolantonio et al., 2013). The beneficial effects of carvedilol might be due to the activation of β-arrestin-mediated transactivation of EGFR that exhibits cardioprotective effects (Noma et al., 2007). These findings show that β-arrestin-biased β-blocker may provide an increased therapeutic benefit compared with unbiased β-blockers.

## Metoprolol

Metoprolol is reported as a biased ligand that specifically induces a G protein-independent and GRK5/β-arrestin2-dependent pathway (Nakaya et al., 2012). Besides receptor regulation, GRKs and β-arrestins have roles in the modulation of cellular signaling via βARs independently of G protein activation. While carvedilol (Wisler et al., 2007; Tzingounis et al., 2010) and alprenolol (Kim et al., 2008) activate intracellular signaling through βARs in the β-arrestin-dependent manner (Table 3). In addition, carvedilol and alprenolol are able to transactivate EGFR, resulting in ERK1/2 activation, whereas metoprolol does not (Nakaya et al., 2012). Administration of metoprolol to mice induced cardiac fibrosis, resulting in a decrease of diastolic function (Nakaya et al., 2012). This fibrotic pathway is mediated through the β_1_AR, which is dependent on β-arrestin2 and GRK5 and is unrelated to G protein action (Table 3). Moreover, metoprolol increases the expression of profibrotic factors, leading to the activation of cardiac fibroblasts, eventually inducing fibrosis (Nakaya et al., 2012).

GRK5, but not GRK6, is necessary for the G protein-independent and β-arrestin2-dependent cardiac fibrosis by metoprolol. Different GRK isoforms phosphorylate distinct sites of βAR to initiate β-arrestin-biased signaling (Violin et al., 2006). For example, inhibition of GRK5 or GRK6 attenuates β-arrestin-mediated ERK1/2 activation following β_1_AR stimulation. Phosphorylation of β_1_AR is not affected by inhibition of GRK2 or GRK3 (Noma et al., 2007; Kim et al., 2008). Therefore, phosphorylation of βARs by different GRK isoforms together with β-arrestin binding is crucial in initiating receptor signaling toward G protein-dependent and β-arrestin-dependent signal transductions. Although metoprolol causes cardiac fibrosis, it remains useful in the management of HF patients (Kukin et al., 1999) since metoprolol attenuates the effect of catecholamine overstimulation in the patients’ hearts and metoprolol-induced fibrosis is neglectable compared with βAR-induced fibrosis (Nuamnaichati et al., 2018) and HF-induced fibrosis.

**FIGURE 1:**
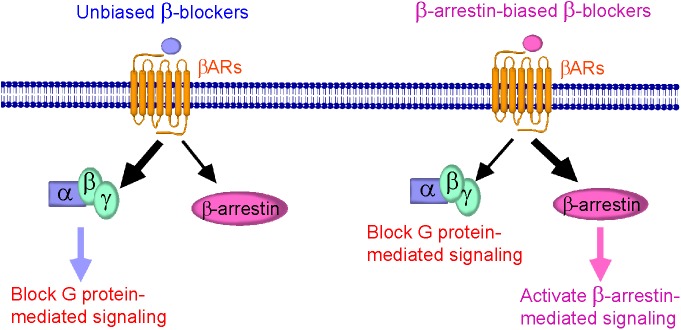
Schematic diagram representing β-blockers mediated β-arrestin-biased signaling. (Left) Binding of unbiased (classical) β-blockers (blue) to βARs resulted in an unbiased response inhibition of G protein-mediated signaling. (Right) Binding of β-arrestin-biased β-blockers (pink) to βARs stabilizes the receptor into a distinct conformation that preferably activates β-arrestin-mediated signaling, resulting in cardioprotection, and is also able to block G protein-mediated signaling.

Understanding of the cellular signaling pathway of β-arrestin-biased β-blockers is important for the development of novel β-blockers that primarily target β-arrestin-mediated βAR signaling with cardioprotective effects. The discovery of other β-arrestin-biased β-blockers (Figure 1) not only provides additional pieces of evidence for their beneficial therapeutic effects, but also helps to design next-generation β-blockers for HF treatment.

## Conclusion

βAR desensitization and overstimulation is the pathological hallmark of HF. Although GRKs play an important role in βAR desensitization, the involvement of individual GRK isoform in the development of a failing heart is not fully understood. Expression and activity of GRKs, especially GRK2, is significantly increased in the failing heart. Thus, inhibition of GRK activity via βARKct gene therapy, synthetic GRK inhibitors, and small peptide GRK inhibitors, represents promising therapeutic approaches for HF treatment. Although GRK2 inhibitors improve cardiac functions in various animal models of HF, these inhibitors have not yet been tested in clinical studies. β-Arrestins are also involved in the regulation of cardiac functions in normal and failing heart. However, the specific contribution of each β-arrestin isoform that plays a role in the development of a failing heart in patients with HF remains to be clarified. Based on the physiological and pathological functions of GRKs and β-arrestins in the heart, both could be candidates for novel theranostic strategies for HF treatment. Cavedilol, alprenolol, and nebivolol are identified as β-arrestin-biased β-blockers that are able to activate β-arrestin-mediated signaling while blocking G protein-mediated signaling, providing cardioprotection. These β-arrestin-biased β-blockers may exhibit distinct pharmacological effects relative to their unbiased counterparts; however, the clinical outcome of these β-blockers remains to be elucidated. Elucidation of the signaling mechanisms of β-arrestin-biased β-blockers will facilitate our understanding and could lead to the discovery of new β-blockers with fewer side effects while providing effective therapy for HF patients.

## Author Contributions

SM and WP mainly wrote the manuscript. HK edited this manuscript.

## Conflict of Interest Statement

The authors declare that the research was conducted in the absence of any commercial or financial relationships that could be construed as a potential conflict of interest.
